# REPeated mAgnetic resonance Image-guided stereotactic body Radiotherapy (MRIg-reSBRT) for oligometastatic patients: REPAIR, a mono-institutional retrospective study

**DOI:** 10.1186/s13014-024-02445-2

**Published:** 2024-04-26

**Authors:** Giuditta Chiloiro, Giulia Panza, Luca Boldrini, Angela Romano, Lorenzo Placidi, Matteo Nardini, Matteo Galetto, Claudio Votta, Maura Campitelli, Francesco Cellini, Mariangela Massaccesi, Maria Antonietta Gambacorta

**Affiliations:** 1 Fondazione Policlinico Universitario Agostino Gemelli, IRCSS, Rome, Italy; 2 Università Cattolica del Sacro Cuore, Rome, Italy

**Keywords:** MR guided- Radiotherapy, Oligometastatic disease

## Abstract

**Background:**

Oligo-progression or further recurrence is an open issue in the multi-integrated management of oligometastatic disease (OMD). Re-irradiation with stereotactic body radiotherapy (re-SBRT) technique could represent a valuable treatment option to improve OMD clinical outcomes. MRI-guided allows real-time visualization of the target volumes and online adaptive radiotherapy (oART). The aim of this retrospective study is to evaluate the efficacy and toxicity profile of MRI-guided repeated SBRT (MRIg-reSBRT) in the OMD setting and propose a re-SBRT classification.

**Methods:**

We retrospectively analyzed patients (pts) with recurrent liver metastases or abdominal metastatic lesions between 1 and 5 centimeters from liver candidate to MRIg-reSBRT showing geometric overlap between the different SBRT courses and assessing whether they were in field (type 1) or not (type 2).

**Results:**

Eighteen pts completed MRIg-reSBRT course for 25 metastatic hepatic/perihepatic lesions from July 2019 to January 2020. A total of 20 SBRT courses: 15 Type 1 re-SBRT (75%) and 5 Type 2 re-SBRT (25%) was delivered. Mean interval between the first SBRT and MRIg-reSBRT was 8,6 months. Mean prescribed dose for the first treatment was 43 Gy (range 24–50 Gy, mean BED_α/β10_=93), while 41 Gy (range 16–50 Gy, mean BED_α/β10_=92) for MRIg-reSBRT. Average liver dose was 3,9 Gy (range 1–10 Gy) and 3,7 Gy (range 1,6–8 Gy) for the first SBRT and MRIg-reSBRT, respectively. No acute or late toxicities were reported at a median follow-up of 10,7 months. The 1-year OS and PFS was 73,08% and 50%, respectively. Overall Clinical Benefit was 54%.

**Conclusions:**

MRIg-reSBRT could be considered an effective and safe option in the multi-integrated treatment of OMD.

## Introduction

The prognosis of oligometastatic disease (OMD) has improved in terms of overall survival (OS) and progression free survival (PFS) in recent years, mainly due to multi-integrated treatment approaches combining novel systemic and local therapies, such as precision radiotherapy (RT) [1–10].

The liver is one of the most common sites of metastases in patients with OMD from almost all types of cancer, and particularly from gastrointestinal, breast and gynecological tumors.

Metastatic liver disease significantly affects therapeutic strategies and organs tolerance considering systemic therapies, surgery or radiotherapy [11]. 

This has led radiation oncologists to increasingly evaluate patients with relapse or oligo-progression disease after previous RT.

In this clinical scenario, re-irradiation with radical intent may be an option in the multi-integrated treatment management of OMD, aiming to increase the efficacy of targeted therapies, immunotherapy or chemotherapy and improve local control (LC) and PFS [12, 13]. 

Despite its wide use in clinical activity, there is no commonly agreed definition of “re-irradiation”. Recently, a Delphi consensus by Andratschke et al., proposed a universally applicable definition of re-irradiation by standardizing a nomenclature to describe different clinical scenarios [14].

In this setting, Stereotactic Body Radiotherapy (SBRT) could be an effective and repeatable local treatment option for patients with OMD [15, 16]. 

Re-irradiation based on SBRT repetition (re-SBRT) has been successfully reported for recurrences in lung, head and neck, pancreatic cancers, prostate cancer and bone metastases [17–24], while only few studies have evaluated the safety and efficacy of re-irradiation in patients with hepatocellular carcinoma (HCC) recurrences and liver malignancies [25–28].

Furthermore, there is no consistent evidence in the literature to support the possibility of liver re-irradiation with SBRT in oligometastatic patients. However, a recent study of Willmann et al. described the possibility of repeating SBRT for progressive or persistence oligometastatic lesions [13].

The significant technical advances in precision RT of the last years may allow more efficacious treatment delivery [29, 30]. As an example, the introduction of Magnetic Resonance image guided radiotherapy (MRIgRT*)*, which allows direct visualization of the tumor and the surrounding organs at risk (OARs) throughout treatment delivery, allowing planning target volume (PTV) margin optimization [31–34] and online adaptive radiotherapy (oART), has increased target coverage and OARs dose sparing capabilities [29, 35–37].

The aim of this retrospective mono-institutional study is to evaluate the toxicity profile of Magnetic Resonance Image-guided Repeated SBRT (MRIg-reSBRT) for liver and perihepatic recurrences in patients with OMD.

Furthermore, a tentative classification for the hepatic re-SBRT scenario is here proposed.

## Materials and methods

### Patients selection and treatment characteristics

We retrospectively collected data of oligometastatic patients that already had a previous course of radiotherapy for peritoneal or hepatic metastatic lesions and subsequently underwent to MRIg-reSBRT for oligorecurrence or oligoprogression metastatic hepatic lesion or peritoneal metastatic lesions within 5 centimeters from the liver.

Inclusion criteria were previous course of hepatic SBRT, histological proven solid tumor, adequate performance status (ECOG 0–3), < 5 nodal lesions, salvage surgery or other local therapies not feasible. The classification of oligometastatic patients by Guckenberger et al. has been used for the classification of OMD patients in our study [2]. 

Absolute contraindications to MRIg-reSBRT were claustrophobia, metal prostheses and/or non-MR compatible medical devices, clinical conditions that did not permit maintaining the supine position and/or actively collaborating with the treatment phases.

All the patients underwent a 0.35 T MRI simulation on the MRIdian system (ViewRay Inc. USA), by acquiring25 seconds or 17 s True Fast Imaging (TRUFI) MR scans in breath-hold inspiration or expiration (BH) or in free breathing (FB), depending on patients’ compliance to gating protocols [38, 39].

Online 4–8 frames per second (fps) cine-MRI allows direct study of target gating by visualizing the lesion and its movement during different phases of respiration. The cine-MRI therefore enables direct assessment of patient compliance, guiding the choice of the most appropriate sequences, the boundary margins and the percentage of region of interest (ROI) accepted as outside the clinically established boundary [38–40].

The gross tumour volume (GTV) was defined on 0.35 T MRI simulation scan and the PTV was obtained by adding a 3- or 5-mm isotropic margin from GTV, depending on the radiological characteristics of the lesions, clinical judgment and patients’ gating compliance.

Re-SBRT dose-fractionation schedule was tailored on the tumor size, location, and distance to sensible OARs (such as small and large bowel, duodenum, and stomach) to achieve an optimal balance between effective radiation treatment and minimizing toxicity.

Total doses were prescribed to the 80% isodose or to the Dmean covering the 95% of the PTV, according to clinical judgement.

Biologically effective dose (BED) was also calculated for the dose prescription considering an α/β value of 10 for the tumor.

Treatment plans were calculated on the dedicated MRIdian Treatment Planning System, using 6 MV beams with a Intensity Modulated Radiotherapy (IMRT) step and shoot VMAT-like approach [41]. In the absence of well-defined dose constraints for abdominal re-irradiation, dose constraints for OARs were primarily evaluated according to the UK SABR Consortium consensus for normal tissue constraints [42].

A rigid registration was performed between the primary MRI of both SBRT courses for contours transfer and dose accumulation. The cumulative equivalent dose in 2 Gy fraction (EQD2) to high-risk OARs between the two courses of SBRT was assessed to esteem the theoretical safety of the retreatment [42–45].

However, if these dose constraints could not be met, a dose reduction was considered and PTV coverage of less than 95% was accepted for lesions for which the dose constraints for OARs could not be met in the summation plan [35, 39].

Delivery was based on the treatment parameters set during the simulation phase and applied to daily cine-MRI which allows direct gating to be performed by stopping the radiation beam when a percentage of the user defined region of interest (ROI%), exceeds a tolerance region, defined as “boundary” [46],.

Starting from the Delphi consensus that describes four categories of re-irradiation scenario [14], we propose a classification for our cases of re-SBRT. The Authors defined Type 1 re-irradiation as “any new course of radiotherapy that has a geometrical overlap, without specific cutoffs isodoses, with the previous irradiated volume”; and Type 2 as “new course without volumes overlap but with toxicity concerns due to cumulative dose to OARs” [14].

Given these two definitions, we attempted to evaluate the proximity between the two SBRT courses, by considering the 2 Gy and 50% isodoses of both SBRT plans.

These isodoses lines were converted into contours and transposed from the first SBRT plan to the planning MRI of MRIg-reSBRT and then the “overlapping volume” was identified using the intersection boolean operator, allowing to measure it in cubic centimeters (cc).

The intersection between the 2 Gy isodoses of first SBRT course (Iso2Gy I) and the re-SBRT (Iso2Gy-re) created an overlap volume (Ov2Gy) in the liver. Instead, the Ov50% is determined by the intersection of at least 50% isodose of first SBRT course (Iso50% I) and re-SBRT ones (Iso50%-re) within the liver.

**Fig. 1:**
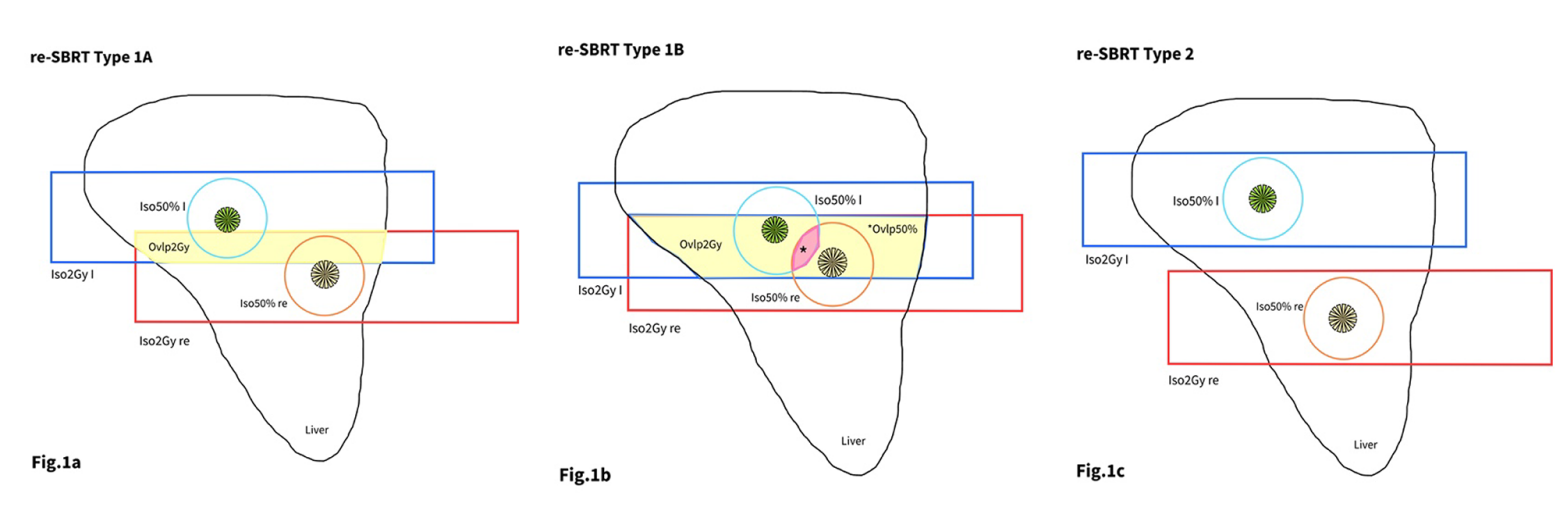
Classification of MRg-re-SBRT Type Rectangle lines are the 2 Gy’s isodoses of the first SBRT course (Iso2Gy I; blue line) and the re-SBRT (Iso2Gy-re; red line), representing field of both treatments. Circle lines are the 50% isodoses (light blue and orange contour) of dose prescription. The Ovlp2Gy volume (yellow volume) is made by the intersection of Iso2Gy I (blue line) and Iso2Gy-re (red line) within the Liver. Instead, the Ovlp50% (pink volume) created by the intersection of the 50% isodoses of first SBRT courses, the Iso50%I (blue line) and re-SBRT, Iso50%-re (orange line) within the Liver. Figure **1a**: MRg-re SBRT Type 1A. Overlap between the Iso2Gy and the Iso2Gy-re. Figure **1b**: MRg-re SBRT Type 1B. Overlap between the Iso50%I and the Iso50%-re. Figure **1c**: MRg-re SBRT Type 2. No geometrical overlap

**Fig. 2:**
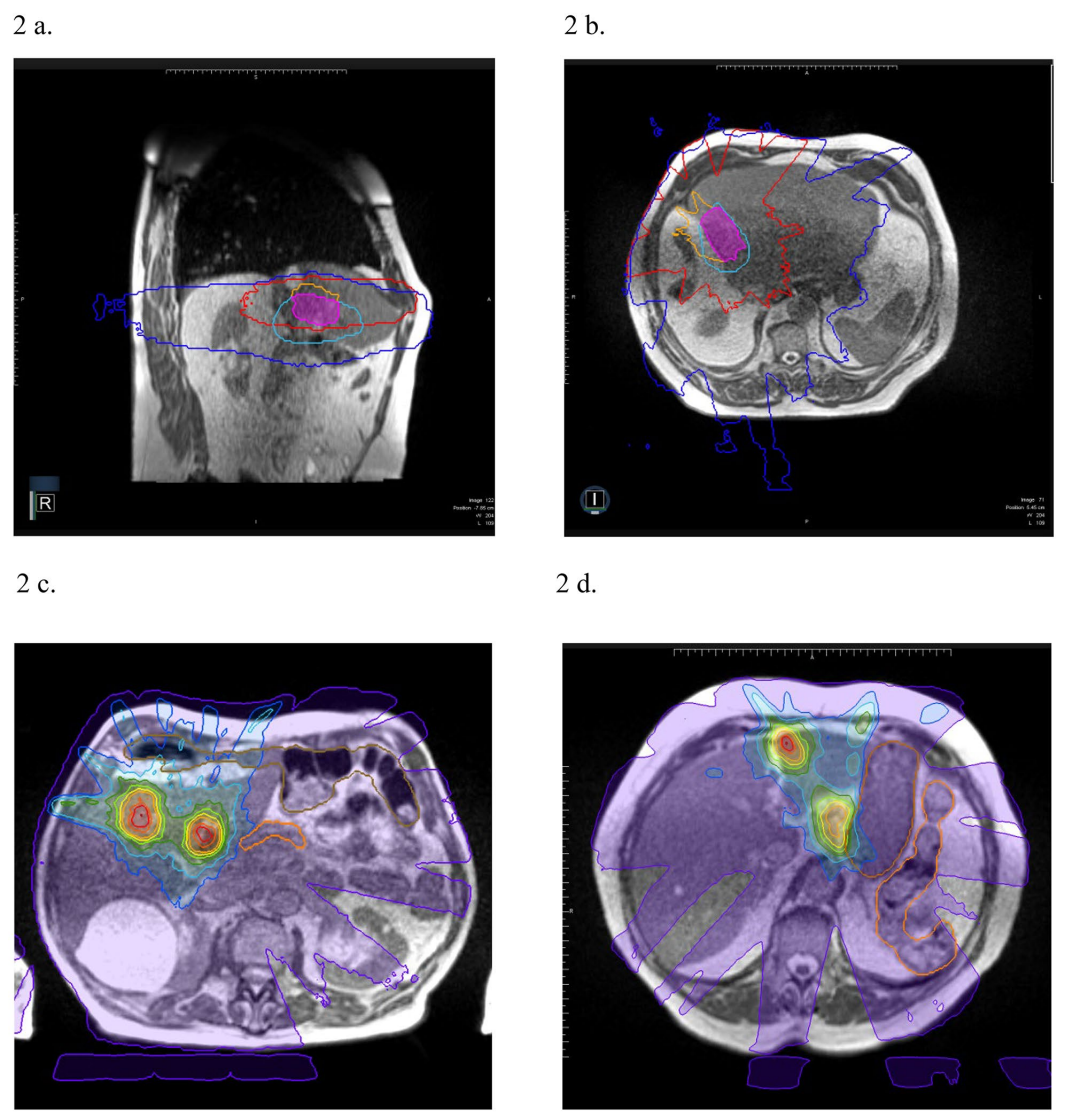
Real example of classification of type 1B re-SBRT and color wash of dose distribution Fig. **2a** and **2b**: Axial and sagittal view of intersection between isodoses lines. Red lines and blue lines represent the 2 Gy’s isodoses of re-SBRT and first SBRT respectively, while the orange line and light blue line represent the 50% isodoses of re-SBRT and first SBRT respectively. Pink volume represent the obverlap volume between the 50% isodoses Fig. **2c** and **2d**: Axial MR scan with color wash dose distribution of type 1B re-SBRT

We classify MRIg-reSBRT as type 1 A when Ov2Gy is created; type 1B in the case of Ov50%; and type 2 when neither Ov2Gy, nor Ov50% can be generated (see Table 1; Figs. 1 and 2).

**Table 1:** Our propose of classification for MRIg-reSBRT

Re-SBRT type	Ov2Gy (cc)	Ov50%
1 A	Yes	No
1B	Yes	Yes
2	No	Not applicable

Type of Re-irradiation:

- Type 1 A: Overlap between the 2 Gy isodoses of both SBRT courses, the Iso2Gy and the Iso2Gy-re.

- Type 1B: Overlap between the 50% isodoses of both SBRT, the Iso50%I and the Iso50%-re

- Type 2: No overlap between the 2 Gy isodoses of both SBRT courses

### Statistical analysis

Descriptive statistics was used to analyze patients, tumor and treatment characteristics.

All toxicities observed within 90 days after treatment were considered acute, while following events were defined as late toxicities.

Acute and late toxicities were assessed using the Common Terminology Criteria for Adverse Events (CTCAE) scale version 5.0 [47]. We investigated on radiation-induced liver disease (RILD), fatigue and gastrointestinal toxicity.

Overall response rate (ORR) and clinical benefit (CB) were assessed 12 months from the end of MRIg-reSBRT.

PFS and Overall Survival (OS) 12 months after MRIg-reSBRT were calculated using the Kaplan Meier curves.

## Results

Eighteen patients completed the prescribed course of MRIg-reSBRT from July 2019 to January 2020.

Seven of them had two metastases at the time of MRIg-reSBRT treatment, while the remaining patients had a single metastasis.

The total number of treated metastatic lesions was 25, with a median interval of 8,5 months (range 2–28 months) between the two treatment courses.

All patients (89%) performed MRI-guided radiotherapy for 22 metastatic lesions, except for two patients (11%).

Two patients underwent to a Transarterial chemoembolization (TACE) for primary HCC lesion, then they performed re-SBRT for hepatic oligorecurrence lesions.

The most common primary tumor were gynecological cancers (42,30%) followed by colorectal cancer (23,1%) and HCC (7,7%). 80% of the irradiated site were liver lesions (*n* = 20) and the remaining 20% were perihepatic peritoneal carcinosis nodules (*n* = 5).

Patients and treatment characteristics are summarized in the Table 2.

**Table 2:** Summary of population treated with MRg-reSBRT

Characteristic	*n* (%)
Age, median (range)	69 (46–87)
Pts (lesion)	18 (25)
Sex	
Male Female	9 (50%) 9 (50%)
Primitive site of tumor	
Colorectal cancer Gynecologist cancer (ovary, uterine) Hepatocellular carcinoma Others	4 (23,1%) 8 (42,30%) 2 (7,7%) 4 (26,9%)
Irradiated metastatic Lesion reSBRT = 25	
Liver Perihepatic abdominal carcinosis	20(80%) 5(20%)
Timing between I and re-SBRT, months, mean (range)	8,5 (2–28)
Oligometastatic disease classification	
Metachronous oligorecurrence Metachronous oligoprogression Induced oligo persistence	4 (22,2%) 12 (66,6%) 2 (11,2%)
Gating treatment for lesion	
Breath hold inspiration Free breathing	18(70%) 7 (30%)
PTV volume (cm3), mean (range)	13,3 (3,6–34,9)
PTV Dose I SBRT liver, median dose (range), mean BED _α/β10_	43,1 (24–50), 95
PTV Dose reSBRT liver, median dose (range), mean BED _α/β10_	41,3 Gy (16–50), 93
Liver dose reSBRT (Gy), mean dose (range), mean EQD2	3,7 Gy (1,6–8 Gy), 3
Mean Follow up, months (range)	11 (3–34)
Toxicity at the mean Follow up	
GI Grade 0–1 GI Grade 2	0 0

Median age at MRIg-reSBRT was 69 years (range 46–87 years). The mean follow-up time was 11 months (range 3–34).

Table 3 reports our classification type of re-SBRT for each patient and the relative re-irradiation data. The mean Ov2Gy was 625,3 cc (range 0-2098 cc).

**Table 3:** Classification of re-irradiation type with defining the overlap between two courses of SBRT and dosimetric characteristics of irradiated lesions per patients

Pts (1–18) (*n*° of Gtvs)	Re-SBRT type	Ovlp2Gy (cc)	Ovlp50% (cc)	Liver Volume I SBRT (cc)	Liver Volume re-SBRT (cc)	Re-SBRT GTV volume (cc)	I-SBRT total dose/ dose for fx (Gy)	re-SBRT total dose/ dose for fx (Gy)	Prescription isodoses	Re-SBRTGTVs coverage Dmean (Gy)	BED_10__dmean	Timing I- reSBRT (months)
1 (2*)	1 A	873	0	1290	1285	8	24/8	24/8	Target mean	24	43	7
2	1 B	917	7,6	1162	1021	19	50/10	40/8	80%	50	99	16
3	1 A	1305	0	1750	1630	5	35/7	35/7	Target mean	35	59	2
4	1 A	205	0	1138	1096	12,7	40/8	50/10	80%	47	87,7	2
5	1 B	1053	65	1671	1672	16,7	50/10	16/4	Target mean	24	35	18
6 (2*)	1 A	488,5	0	1428	1321	10,8	50/10	40/8	80%	47,7	88,5	5
7	2	27,8	0	1208	1106	2,2	50/10	50/10	80%	59	123	11
8 (2°)	1 A	2098	0	1352	1346	1,9	40/8	50/10	80%	57,3	116,4	3
		2098	0	1352	1346	5,3	40/8	50/10	80%	57,1	116	
9 (2*)	1 A	602	0	1403	1349	3,6	50/10	40/8	80%	50	99	2
10	2	0	0	1610	2009	9,1	50/10	50/10	80%	58	120	5
11	2	0	0	1017	1017	2,2	40/8	40/8	80%	46,25	83,25	3
12	1 B	380	1	1128	1131	1,5	35/7	35/7	80%	43,65	71,46	6
13	1 B	962,4	2	1112,7	1266,9	1,7	50/10	50/10	80%	64,62	133,36	18
14	1 A	417	0	975	1029	1,8	50/10	35/7	Target mean	35,6	59,2	5
15 (2*)	1 B	1448	5	1619,9	1647	12,5	40/8	50/10	80%	62,92	132,38	4
16	2	11	0	1219	1115	2,6	50/10	40/8	80%	51	93,06	28
17 (2*)	2	17,8	0	1127	967	3,1	50/10	50/10	80%	50,6	100	12
18 (2°)	1 B	450	3	1198,4	1303	2	40/8	40/8	80%	48,76	88,5	6
		450	3	1198,4	1303	2,9	40/8	35/7	80%	37,7	61,5	

Table 3: * Two lesions treated as unique GTV sum with dose prescription to one PTV sum. °Two separated GTVs treated with two different dose prescription to PTVs or two different plans

In two cases we did not report any field overlap and classified them as Type 2 re-SBRT. Instead, we classified 15 courses of re-SBRT as Type 1, with median geometric overlap between the 2 Gy’s isodoses of both courses of 861 cc (range 205–2098).

In three cases we obtained minimal field overlap (range 11–27,8 cc), so we decided to also classify these cases as Type 2 re-SBRT. In 7 cases (35%) we also reported superposition between the 50% isodoses of the dose prescription (range 1–65 cc), classifying them as type 1B re-SBRT, besides type 1A re-SBRT in those were not. Overall, we reported 5 Type 2 re-SBRT (25%) and 15 Type 1 re-SBRT (75%), and among this we distinguished 8 Type 1B re-SBRT (40%) and 7 Type 1 A re-SBRT (35%).

Median PTV margin was 3,6 (range 3–5 mm). Overall, no PTV overlap was observed.

The mean GTV volume of MRIg-reSBRT was 6,8 cc (range 1,5–19,9), with a median PTV of 13,4 cc (range 3,6–34,9).

The mean normal liver volume (defined as liver less GTV) of the two courses of SBRT was 1970 (926–1198) cc and 1294 (967–2009) cc, respectively.

The mean prescribed dose for the first SBRT was 43 Gy (range 24–50 Gy, mean BED10 = 93) and 41 Gy (range 16–50 Gy, mean BED10 = 92) for MRIg-reSBRT, in five fraction.

In five cases original prescription dose was reduced due to the proximity of OARs or to a time less than six months between the two courses. For one hepatic lesion we have opted for 16 Gy in four fractions because of the size of PTV (29 cm^3^) and proximity to OARs (small bowel).

We prescribed 24 Gy in three fractions for two hepatic lesions adjacent to each other and between the previous SBRT. In three cases (one hepatic lesion and two carcinosis nodules) we prescribed 35 Gy in five fractions to maintain the dose constraints to the duodenum (D_0,1 cc_=35 Gy).

Treatment was prescribed to the 80% isodose for 20 lesions (80%), and to Dmean, covering the 95% of the PTV, in the remaining five (20%).

The mean PTV D2 and mean PTV D98 of MRIg-reSBRT were 51 Gy (range 16,5–67,5) and 41 Gy (range 15–59,5).

The average mean liver dose was 3,9 Gy (range 1–10 Gy; median EQD2 2,9 Gy) and 3,7 Gy (range 1,6–8 Gy; median EQD2 3 Gy) for the first SBRT and MRIg-SBRT, respectively.

Overall, 17 lesions (70%) were treated in inspiration BH.

Online adaptive radiotherapy was applied in five treatment plans, for seven different lesions (five liver metastases and two carcinomatosis lesions). Overall, 16 fractions out of 25 fractions were adapted.

In one perihepatic carcinomatosis case, treatment plan was online adapted every single day (5/5 fractions), while oART was performed at least one fraction for day in the other four cases.

All patients completed the foreseen MRIg-reSBRT course without interruptions and no toxicity of any grade was observed at a median follow-up of 11 months after MRIg-reSBRT.

More specifically, no cases of radiation-induced liver disease (RILD) or fatigue have been reported.

After the MRIg-reSBRT course, 16 patients have been following systemic therapies: chemotherapy (27,7%) immunotherapy (44,6%) or both (27,7%).

At 1 year, OS and PFS were 85% and 55%, respectively, with an ORR and CB both of 54%.

## Discussion

As far as the authors know, this is the first experience reporting re-irradiation SBRT safety outcomes in abdominal OMD setting. Limited evidence is reported in literature regarding the possibility to re-SBRT for in- and out-of-field liver recurrences and this is mainly related to HCC [48].

Lo et al. reported repeated liver SBRT for 14 patients with HCC with a median EQD2_10_ of 60 Gy, observing a RILD in one patient and three patients showing a progression of the Child Pugh score after the re-SBRT [25].

Gkika et al. reported 24 patients who underwent re-SBRT for HCC recurrences “in- and out-of-field” after previous SBRT, with a median prescribed dose for re-SBRT of 48 Gy (range 27–66 Gy, EQD2_10_ = 71) and a median mean liver dose (Dmean, liver) of 6 Gy (range 1–25, EQD2_2_ = 7 Gy) for the first SBRT and 10 Gy (range 1–63 Gy, EQD2_2_ = 9 Gy) for the re-SBRT [49]. Other studies reported re-irradiation using 3D-RT with a moderate hypofractionation (1.8–3 Gy) [48, 50].

Besides the limited evidence, another fundamental issue is the exact definition of re-irradiation in the different possible scenarios.

The term “re-irradiation” includes indeed different conditions, as recently proposed by a Delphi consensus performed by Andratschke and colleagues. They standardized a nomenclature to describe different re-irradiation clinical scenario.

The classification proposed in the study aimed to categorize re-irradiation scenarios based on the overlap of radiation fields and potential toxicity concerns.

Based on their classification [14] we here proposed a MRIgre-SBRT classification, taking in consideration the 2 Gy isodoses, that represent the radiation beam path through the patient, and the 50% isodoses representing instead the high dose region where the target is located.

In almost all cases (75%) there was a superposition between the 2 Gy isodoses of the two SBRT courses, meaning a geometrical overlap between the two field of radiation fields (Type 1A re-SBRT).

Only five cases did not have this overlap, and we considered them as “out of field” re-SBRT. Nevertheless, due to the concerning proximity of OARs, such as duodenum and other gastrointestinal organs, we included them in our study as Type 2 re-SBRT [14]. 

For this type of re-SBRT, estimating the distance between GTVs and OARs and the time elapsed between the two SBRT courses seems to be more relevant than dosimetric variables alone.

An overlap between the 50% isodoses of the prescribed dose (type 1B re-SBRT) was observed in 35% of the reported cases, but without volumetric overlap between the GTVs. This could indicate an increased risk of toxicity to surrounding OARs, given the proximity of the two lesions.

Our classification of re-irradiation scenarios for OMD focuses on geometric overlap assessment, toxicity concerns and dosimetric evaluation. By examining the spatial relationship between radiation fields and analysing isodose overlap, the classification contributes to understanding treatment volume interactions and guiding dose distribution. It also considers cumulative dose to OARs to address potential toxicity risks with repeated SBRT treatments. This systematic approach improves treatment planning and patient safety and contributes to the standardization of re-irradiation terminology and reporting in the literature.

As no toxicity was observed at all, it was not possible for us to explore a volumetric overlap threshold to hypothesize a dose constraint and define a further subclassification of re-SBRT, especially considering the parallel type architecture of the liver [51].

These positive results may also be related to the applied oART approach, which probably improved the therapeutic ratio of MRIgSBRT.

MRIgRT can reduce the daily uncertainties providing direct visualization of the tumor and surrounding tissue allowing PTV margins’ reduction, leading to a reduction of irradiated healthy liver volume, optimizing the dose distribution on a daily basis if necessary.

Also, MRI-guidance provides real-time visualization of the liver and the targeted lesion, offering enhanced precision in treatment delivery. This is especially crucial in cases of reirradiation, where healthy surrounding tissue may have already received radiation. The superior soft tissue contrast of MRI allows for accurate target delineation and adaptation, minimizing the risk of damage to critical structures. Additionally, daily adaptation based on the patient’s anatomical variations allows for dynamic treatment planning, ensuring that the radiation is delivered with the utmost accuracy [33]. This is particularly advantageous in the context of hepatic lesions, which can be subject to respiratory motion and other anatomical changes [37]. 

In addition, the non-invasive nature of MRI-guided radiation therapy minimizes the need for invasive fiducial markers or surrogates, reducing patient discomfort and potential complications.

Furthermore, the few available studies about SBRT re-irradiation involve small patients groups with no clear information on the maximum dose administered to the OARs and no uniform constraints for this scenario [44] or in particular for MRI guided treatment plan.

For target lesions close to the gastrointestinal tract, it has been suggested to keep the cumulative dose to the bowel under 98 Gy (EQD2) and V15 Gy of the second SBRT course below 120 cc, as late complications (e.g. small bowel obstruction, persistent diarrhea and fistulas), are observed in 20-30% of patients when doses exceed 100–110 Gy (EQD2) for a maximum volume of 10 cc [32, 36].

All types of MRg-reSBRT were well tolerated in our experience, even those where a 50% isodose overlap was observed.

However, in these cases, we often have to reduce the prescription dose to preserve gastrointestinal OARs that have already received previous dose, especially if the time between the two courses was less than six months as we report above.

The interval between previous SBRT and MRI-guided reSBRT influenced the dosing decisions, particularly to manage the cumulative dose and mitigate toxicity risks. Each schedule was customized considering tumor specifics OARs constraints, and clinical context. Higher doses per fraction (8 to 10) were applied to smaller tumors away from critical structures for better local control, while lower dose per fraction (5 to 7) were used for larger tumors or those close to OARs to reduce toxicity risks.

Considering clinical outcome, we obtained at least a modest clinical benefit. This slight upward trend, considering the small sample size, seems to be in line with the literature data for oligometastatic patients [52, 53].

Our results should be evaluated in the context of the limitations of this study, such as the small sample size, and the non-randomized retrospective single-center nature of the study. Another limitation is the relatively short follow-up period, which does not allow a proper assessment long-term outcomes and late-onset toxicities.

In addition, our patient cohort consisted of individuals in a clinical setting characterized by oligometastatic disease, which is inherently heterogeneous in terms of disease presentation and treatment response. These limitations underscore the importance of caution when extrapolating our findings to larger populations. They also underscore the need for larger, prospective, multicenter studies to validate our findings [54, 55]. 

Furthermore, a patient selection bias may also have contributed to these favorable results: none of the patients had impaired liver function prior to RT, although all patients had undergone a long-term course of various systemic therapies.

Nevertheless, this aspect reflects the usual clinical presentation of this category of patients, with the ones affected by liver metastases tending to have relatively well-preserved liver function when compared to HCC cases, who often suffer from severe underlying liver diseases such as viral hepatitis, alcohol induced cirrhosis or non-alcoholic steatohepatitis.

Moreover, the results of our study of MRIg-reSBRT for OMD not only confirm its efficacy and safety, but also highlight significant implications and future directions for radiation oncology. The integration of advanced technologies such as MRI guidance into clinical practice has proven to be pivotal in improving the precision and accuracy of treatment, particularly in scenarios involving repeated SBRT. This highlights the critical role of technological advances in modern oncology practice. Future research should consider the synergy of MRI guidance with innovative techniques such as artificial intelligence and radiomics to further push the boundaries of treatment precision and outcomes. In addition, the adoption of personalized treatment strategies tailored to individual patient characteristics and tumour biology could optimize therapeutic outcomes in the management of OMD. A thorough evaluation of the cost-effectiveness and resource utilization of MRI-guided technologies compared to conventional treatments is essential for informed healthcare decision-making. In addition, fostering global collaboration and establishing knowledge-sharing platforms are critical to disseminating best practices and innovative approaches across the field. By adopting these strategies, we can improve patient care and refine treatment protocols for the management of oligometastatic disease, ensuring that our approaches are both scientifically sound and clinically relevant.

## Conclusion

Both type 1 and type 2 MRIg-reSBRT appear to be an effective and clinically well tolerated integrated option in a multi-treatment strategy for this class of oligoprogressive or oligorecurrence patients of our study.

We believe that estimating the overlap between the highest isodoses (> 50%), PTVs and even evaluating the overlap with OARs coupled with the innovative oART paradigm, may help clinical decision-making regarding dose prescription and safety of retreatment.

MRIg-reSBRT could be considered as a potential salvage option for patients unfit for other interventional approaches in order to improve survival outcome or disease control by extending chemotherapy free survival and could also be integrated with novel targeted therapies or immunotherapy in the near future, considering the overall good tolerability and favorable logistic aspects.

In this regard, prospective randomized trials with larger sample size are necessary to better evaluate the clinical outcomes of this innovative procedure. Standardized guidelines and classification systems for abdominal SBRT re-irradiation are also warranted, to define the most appropriate delivery technique, prescription dose and reliable OARs constraints.

## Data Availability

No datasets were generated or analysed during the current study.

## Abbreviations

OMD: Oligometastatic disease
OS: Overall Survival
PFS: Progression Free Survival
RT: Radiotherapy
LC: Local Control
SBRT: Stereotactic Body Radiotherapy
re-SBRT: SBRT repetition
HCC: Hepatocellular carcinoma
MRIgRT: Magnetic Resonance image guided radiotherapy
OARs: Organs at risk
PTV: Planning target volume
oART: Online adaptive radiotherapy
MRIg-reSBRT: Magnetic Resonance Image-guided Repeated SBRT
TRUFI: True Fast Imaging
FB: Free breathing
BH: Breath-hold
GTV: Gross tumour volume
BED: Biologically effective dose
IMRT: Intensity Modulated Radiotherapy
EQD2: Cumulative equivalent dose in 2 Gy fraction
Iso2Gy: I 2 Gy isodoses of first SBRT course
Iso2Gy-re: 2 Gy isodoses of re-SBRT course
Ov2Gy: 2 Gy overlap volume
Ov50%: 50% overlap volume
Iso50%: I 50% isodose of first SBRT course
Iso50%-re: 50% isodose of re-SBRT course
ORR: Overall response rate
CTCAE: Common Terminology Criteria for Adverse Events
CB: Clinical benefit
TACE: Transarterial chemoembolization
RILD: Radiation-induced liver disease
